# Functional recombinant protein is present in the pre-induction phases of *Pichia pastoris* cultures when grown in bioreactors, but not shake-flasks

**DOI:** 10.1186/s12934-014-0127-y

**Published:** 2014-09-04

**Authors:** Zharain Bawa, Sarah J Routledge, Mohammed Jamshad, Michelle Clare, Debasmita Sarkar, Ian Dickerson, Markus Ganzlin, David R Poyner, Roslyn M Bill

**Affiliations:** School of Life and Health Sciences, Aston University, Aston Triangle, Birmingham, B4 7ET United Kingdom; AstraZeneca Ltd, Alderley Park, Macclesfield, Cheshire, SK10 4TF United Kingdom; School of Biosciences, University of Birmingham, Edgbaston, Birmingham, B15 2TT UK; Department of Neurobiology and Anatomy, University of Rochester Medical Center, Rochester, New York 14642 USA; present address for MG, Lonza Group Ltd, Muenchensteinerstrasse 38, CH-4002 Basel, Switzerland

**Keywords:** *P. pastoris*, Methanol induction, Pre-induction expression, Bioprocess, Adenosine A_2a_ receptor, GPCR, GFP, CD81, CD82, Claudin-1, HRP, CGRP-RCP

## Abstract

**Background:**

*Pichia pastoris* is a widely-used host for recombinant protein production; expression is typically driven by methanol-inducible alcohol oxidase (*AOX*) promoters. Recently this system has become an important source of recombinant G protein-coupled receptors (GPCRs) for structural biology and drug discovery. The influence of diverse culture parameters (such as pH, dissolved oxygen concentration, medium composition, antifoam concentration and culture temperature) on productivity has been investigated for a wide range of recombinant proteins in *P. pastoris*. In contrast, the impact of the pre-induction phases on yield has not been as closely studied. In this study, we examined the pre-induction phases of *P. pastoris* bioreactor cultivations producing three different recombinant proteins: the GPCR, human A_2a_ adenosine receptor (hA_2a_R), green fluorescent protein (GFP) and human calcitonin gene-related peptide receptor component protein (as a GFP fusion protein; hCGRP-RCP-GFP).

**Results:**

Functional hA_2a_R was detected in the pre-induction phases of a 1 L bioreactor cultivation of glycerol-grown *P. pastoris*. In a separate experiment, a glycerol-grown *P. pastoris* strain secreted soluble GFP prior to methanol addition. When glucose, which has been shown to repress *AOX* expression, was the pre-induction carbon source, hA_2a_R and GFP were still produced in the pre-induction phases. Both hA_2a_R and GFP were also produced in methanol-free cultivations; functional protein yields were maintained or increased after depletion of the carbon source. Analysis of the pre-induction phases of 10 L pilot scale cultivations also demonstrated that pre-induction yields were at least maintained after methanol induction, even in the presence of cytotoxic concentrations of methanol. Additional bioreactor data for hCGRP-RCP-GFP and shake-flask data for GFP, horseradish peroxidase (HRP), the human tetraspanins hCD81 and CD82, and the tight-junction protein human claudin-1, demonstrated that bioreactor but not shake-flask cultivations exhibit recombinant protein production in the pre-induction phases of *P. pastoris* cultures.

**Conclusions:**

The production of recombinant hA_2a_R, GFP and hCGRP-RCP-GFP can be detected in bioreactor cultivations prior to methanol induction, while this is not the case for shake-flask cultivations of GFP, HRP, hCD81, hCD82 and human claudin-1. This confirms earlier suggestions of leaky expression from *AOX* promoters, which we report here for both glycerol- and glucose-grown cells in bioreactor cultivations. These findings suggest that the productivity of *AOX*-dependent bioprocesses is not solely dependent on induction by methanol. We conclude that in order to maximize total yields, pre-induction phase cultivation conditions should be optimized, and that increased specific productivity may result in decreased biomass yields.

**Electronic supplementary material:**

The online version of this article (doi:10.1186/s12934-014-0127-y) contains supplementary material, which is available to authorized users.

## Background

*P. pastoris* is a simple eukaryote with a fully sequenced genome [1,2], which can be cultured to high biomass yields without incurring high costs [3]. In its most widely used format, protein production is driven by a very strong, methanol-inducible alcohol oxidase (*AOX*) promoter, which is generally accepted to be tightly regulated [4]. To take full advantage of its attributes, *P. pastoris* must be cultured in controlled bioreactors, rather than shake-flasks, in cultivations that typically comprise several distinct phases. During the first or batch phase, cells grow at their maximum growth rate (μ_max_) until the initial carbon source, typically glycerol, has been depleted. In a subsequent fed-batch phase, the same carbon source is fed continuously with the objective of yielding high pre-induction biomass; during this phase, growth is nutrient limited and a constant specific growth rate, lower than μ_max_, is achieved. A transition phase, when the glycerol feed is stopped and the cells are monitored for glycerol depletion, allows the cells to adapt to low concentrations of inducer (typically methanol); in some cases temperature changes are also applied to facilitate induction at a temperature optimized for a given target protein. Finally, in the induction phase, methanol is added in a controlled manner [5,6] to induce *AOX*-driven recombinant protein production.

Recently *P. pastoris* has become an important source of recombinant G protein-coupled receptors (GPCRs) for structural biology and drug discovery. Drugs targeting GPCRs, which transmit a wide array of signals in different cell types, underpin modern medicine [7]; they account for about 40% of all prescription pharmaceuticals on the market. Relevant structural results are exemplified by the X-ray crystal structure of recombinant human histamine H1 receptor bound to doxepin at 3.1 Å resolution [8] and the 2.7 Å resolution structure of recombinant A_2a_ adenosine receptor (hA_2a_R ) in complex with an antibody Fab fragment that stabilizes an inactive form of the receptor [9].

Numerous studies have examined the influence of parameters such as the temperature and pH of the culture, the amount of dissolved oxygen (DO) in the culture medium and, specifically, the addition of chemical additives and ligands for optimal GPCR production in the pre-induction and induction phases of *P. pastoris* cultivations [4,10–13]. Notably, Singh and colleagues observed active hA_2a_R in bioreactor cultures prior to the methanol feed [14], while this was not apparent in shake flasks [15]. These findings are especially noteworthy because glycerol, glucose, ethanol and acetate have all been shown to support growth of *P. pastoris* cells without inducing the *AOX* promoter [16]. For example, Hellwig and colleagues [17] demonstrated glycerol in the culture medium inhibited production of a recombinant single-chain antibody in mixed feed bioreactor cultures, while Inan and Meagher reported that ethanol and acetate repress *AOX*-driven expression [16].

The first aim of our study was to specifically investigate the pre-induction phases of *P. pastoris* bioreactor cultivations producing recombinant hA_2a_R. We examined a glycosylation-deficient hA_2a_R mutant designed by Fraser [18], who had previously achieved a functional yield of 4 pmol mg^−1^ in shake flask cultures with no pre-induction activity. Prior to methanol induction, we detected baseline production of hA_2a_R confirming that expression driven by the *AOX* promoter is leaky in a glycerol-containing medium. In these cultures the baseline yield of hA_2a_R was not substantially improved in the methanol induction phase and pre-induction yields of hA_2a_R were maintained even at cytotoxic levels of methanol. In order to assess whether these observations were specific to hA_2a_R as the recombinant target protein, two additional strains were investigated; one that secretes the model protein, soluble green fluorescent protein (GFP) and one that secretes the peripheral membrane protein, human calcitonin gene-related peptide receptor component protein (as a fusion protein with GFP; hCGRP-RCP-GFP). When grown on glycerol or glucose as the pre-induction carbon source, GFP (as for hA_2a_R) was produced in the pre-induction phases. Following methanol induction, yields were not substantially improved for glycerol-grown cells, but were increased by a factor of 4 without a corresponding increase in biomass for glucose-grown cells. Pre-induction expression of hCGRP-RCP-GFP was detected in glycerol-grown cells. In contrast, there was no pre-induction expression in shake-flask cultures of strains producing GFP, horseradish peroxidase (HRP), the human tetraspanins hCD81 and CD82 or the tight-junction protein human claudin-1. These data demonstrate that bioreactor but not shake-flask cultivations exhibit recombinant protein production in both glycerol- and glucose-grown pre-induction phases. This highlights the importance of understanding the influence of pre-induction phase cultivation conditions on both recombinant protein yields and *P. pastoris* biomass yields.

## Results

### hA_2a_R is produced in the pre-induction phases of glycerol-grown *P. pastoris* bioreactor cultivations

**Figure 1:**
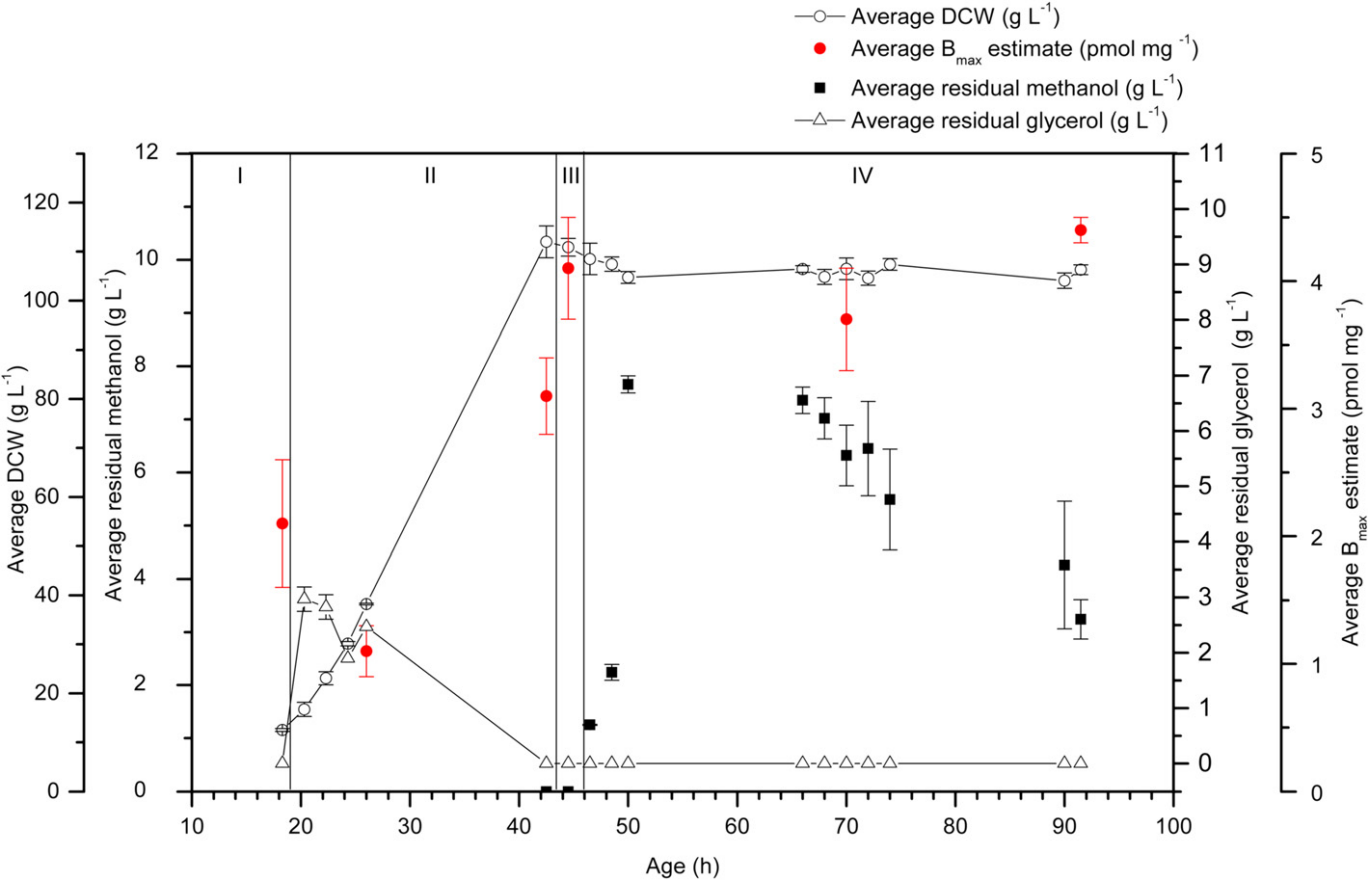
**hA**_**2a**_ **R binding activity is present in all phases of 1 L glycerol-grown** ***P. pastoris*** **bioreactor cultivations.** Duplicate 1 L bioprocesses were analysed for hA_2a_R binding activity (pmol mg^−1^; red circles), DCW (g L^−1^; white circles), residual glycerol concentration (g L^−1^; white triangles) and residual methanol concentration (g L^−1^; black squares). hA_2a_R binding activity was measured in all the phases, including pre-induction phases I and II. The residual glycerol concentration was at its highest during phase II, dropping to zero in phase II and for the duration of the cultivation. DCW increased during phases I and II and plateaued during phases III and IV. Residual methanol ranged from 1.25 – 7.66 g L^−1^ during the induction phase. Measurements were made in triplicate for each culture. The error bars represent standard error of the mean (SEM).

Duplicate 1 L bioreactor cultivations of *P. pastoris* producing a glycosylation-deficient hA_2a_R mutant [18] were examined over a 91 h cultivation period. Since it is established that lowering the temperature to 22°C during induction increases the functional yield of hA_2a_R [13,14,18], the cultivation temperature was lowered during phase III. Figure 1 shows the glycerol batch (I), glycerol fed-batch (II), transition (III) and methanol induction (IV) phases analysed in this study together with the residual glycerol measurements, dry cell weight (DCW) and B_max_ estimates for recombinant hA_2a_R binding activity, as measured by radio-ligand binding with the tritiated antagonist, ZM241385. Residual glycerol peaked during phase II at ~ 2 g L ^−1^ and was not present during the transition and induction phases (III and IV). Binding activity between 1.1 and 3.1 pmol mg^−1^ was measured during the batch and fed-batch phases (I and II), indicating leaky expression. During the transition phase (III), binding activity increased to 4.1 pmol mg^−1^ and during the induction phase (IV) it was 3.7 – 4.4 pmol mg^−1^. The specific yield from the 1 L cultivation was 122.2 pmol g^−1^ and the total yield was 12,986.3 pmol. Notably, the impact of the methanol feed was minimal since the pre-induction specific yield was not substantially increased (Figure 1).

### hA_2a_R is produced in glycerol-grown *P. pastoris* cultivations in the absence of a methanol induction step, while pre-induction binding activity is maintained at pilot scale, even at cytotoxic methanol concentrations

**Figure 2:**
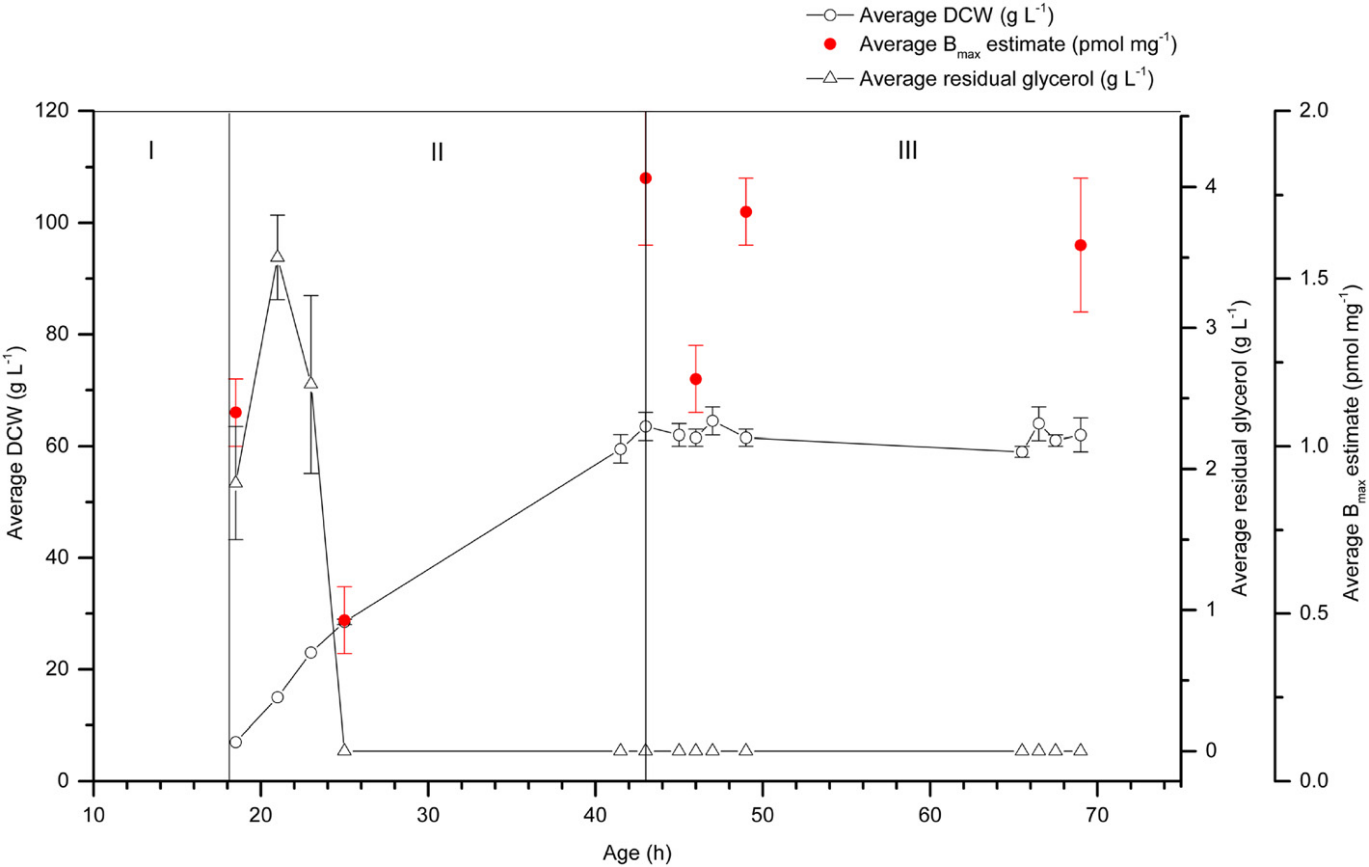
**hA**_**2a**_ **R binding activity is present in all phases of 1 L methanol-free cultivations.** Duplicate 1 L bioprocesses for the same strain cultured previously (Figure 1) were analysed for hA_2a_R binding activity (pmol mg^−1^; red circles), DCW (g; white circles) and residual glycerol concentration (g L^−1^; white triangles) in the absence of methanol induction. hA_2a_R binding activity was measured in all the phases: glycerol batch phase (I); glycerol fed-batch phase (II) and carbon source starvation phase (III). The residual glycerol concentration was at its highest during phase I, dropping to zero in phase II and for the duration of the cultivation. DCW increased during phases I and II and plateaued during phases III. Measurements were made in triplicate. The error bars represent the SEM.

hA_2a_R was also produced in all phases of a completely methanol-free cultivation of glycerol-grown *P. pastoris* (Figure 2). During the fed-batch phase (II), when glycerol was present at 1.9 g L^−1^, the recombinant hA_2a_R yield was 1.1 pmol mg^−1^, reaching a final yield of 1.6 pmol mg^−1^ at the end of the cultivation. The specific yield from the 1 L cultivation was 90.3 pmol g^−1^ and the total yield was 5,598.3 pmol. This was lower than the yield achieved in the corresponding induced culture (Figure 1; 122.2 pmol g^−1^ and 12,986.3 pmol at the end of phase IV) indicating the positive impact of the methanol feed on total yield. The DCW reached a maximum of 62.0 g L^−1^ in contrast to that of 106.3 g L^−1^ for the corresponding induced culture (Figure 1).

To examine whether these findings transferred to larger vessels, two simultaneous pilot scale bioreactor cultivations (10 L starting volume) of *P. pastoris* expressing hA_2a_R were compared following the same batch, fed-batch and transition feeding regimes (Table 1). During the induction phase, different methanol feed profiles were applied: a low methanol feed profile with methanol concentrations maintained below 5.0 g L^−1^ and a higher methanol feed profile with methanol concentrations allowed to exceed 5.0 g L^−1^. A total cultivation time of 91.2 h included the following phases: batch (phase I), fed-batch (phase II), transition (phases IIIA and IIIB) and induction (phase IV and V). An identically-conducted batch phase (I) on 10 g L^−1^ glycerol lasted for 19.1 h for both bioprocesses. At this point, the characteristic DO spike indicated full consumption of the glycerol carbon source and the stirrer speed decreased as a result of the DO control. In the subsequent 20 h fed-batch phase (II), the same exponential, growth rate-limiting glycerol feed profile of 4 g L^−1^ h^−1^ was applied to both bioprocesses, and increased exponentially at a rate of 0.15 h^−1^ for 10 h and then 0.03 h^−1^ until the end of this phase. The transition phase had two different sections (IIIA and IIIB): in phase IIIA, no feed was applied to the cultivations for 1 h; in phase IIIB a constant methanol feed of 4 g L^−1^ h^−1^ was applied. At the end of phase IIIB (47 h), the cultivation temperature was lowered to 22°C and then the induction phase commenced differently for the two cultivations. For both cultivations, methanol was added in an exponential manner. For the low methanol fed cultivation, a total of 2.2 kg of methanol was added and for the high methanol fed cultivation, a total of 3.8 kg methanol was added by the end of the cultivation.

**Table 1:** **Pre-induction yields of hA**_**2a**_ **R are maintained at pilot scale even in the presence of cytotoxic methanol concentrations**

	**Phase**	**Age (h)**	**Residual glycerol (g L** ^**−1**^ **)**	**Residual methanol (g L** ^**−1**^ **)**	**DCW (g L** ^**-1**^ **)**	**Total membrane protein yield in 1 L culture (mg)**	**B**_**max**_ **estimate (pmol mg** ^**−1**^ **)**	**Total hA**_**2a**_ **R yield in 1 L culture (pmol)**	**Specific yield (pmol g** ^**−1**^ **)**
**Low methanol cultivation**	IIIA	42.2	0 (0)	0 (0)	41.91 (0.6)	764.4	4.4 (0.1)	3363.6	80.3
	IIIB	44.4	1.1 (0.2)	0.5 (0.1)	42.10 (0.4)	2791.1	7.1 (0.6)	19816.9	470.7
	IV	66.4	0 (0)	1.0 (0.1)	50.53 (0.5)	4084.4	4.5 (0.2)	18380.0	363.7
	IV	89.0	0 (0)	0 (0)	71.82 (0.2)	7704.4	5.0 (0.2)	38522.2	536.4
**High methanol cultivation**	IIIA	42.2	2.9 (0.1)	0 (0)	46.71 (0.5)	813.3	4.4 (0.2)	3578.7	76.6
	IIIB	44.4	4.2 (0.2)	2.1 (0.2)	46.66 (0.7)	3060.0	4.0 (0.0)	12240.0	262.3
	IV	66.4	1.1 (0.2)	39.1 (9.0)	45.76 (0.5)	3277.8	3.8 (0.1)	12455.5	272.2
	V	88.9	0 (0)	84.7 (0.5)	37.64 (0.8)	1797.8	3.1 (0.1)	5573.1	148.1

The total yield of hA_2a_R (pmol) and the specific yield of hA_2a_R per gram of DCW (pmol g^−1^) were derived from the B_max_ and total membrane protein measurements, as described in the Methods section. Values are calculated per L of culture for the transition and induction phases of the low and high methanol cultivations and are the mean of triplicate determinations, with the standard error of the mean in parentheses. Triplicate measurements were from three different membrane preparations and standard error of the mean is shown in parentheses.

Table 1 shows B_max_ estimates, residual glycerol and methanol concentrations and the amount of biomass generated (DCW and total membrane protein) for both cultivations during the transition and induction phases. The low methanol cultivation produced the highest total and specific yields of hA_2a_R. Even in the absence of methanol, hA_2a_R binding activity was 4.4 pmol mg^−1^ for both the low (SEM = ± 0.1 pmol mg^−1^) and high methanol (SEM = ± 0.2 pmol mg^−1^) cultivations (Table 1). For the low methanol cultivation, in phase IIIB, where a constant methanol feed was initiated and glycerol was still present at 1.1 g L^−1^, functional receptor was detected. In phases IV and V of the low methanol cultivation, no glycerol was present and binding activity was present. For the high methanol cultivation during the transition and induction phases (IIIB and IV), glycerol was present in the culture medium and binding activities were 4.0 and 3.8 pmol mg^−1^, respectively. In phase V, there was no residual glycerol in the culture medium and binding activity was 3.1 pmol mg^−1^.

### GFP is produced in the pre-induction phases of glycerol-grown *P. pastoris* bioreactor cultivations

**Figure 3:**
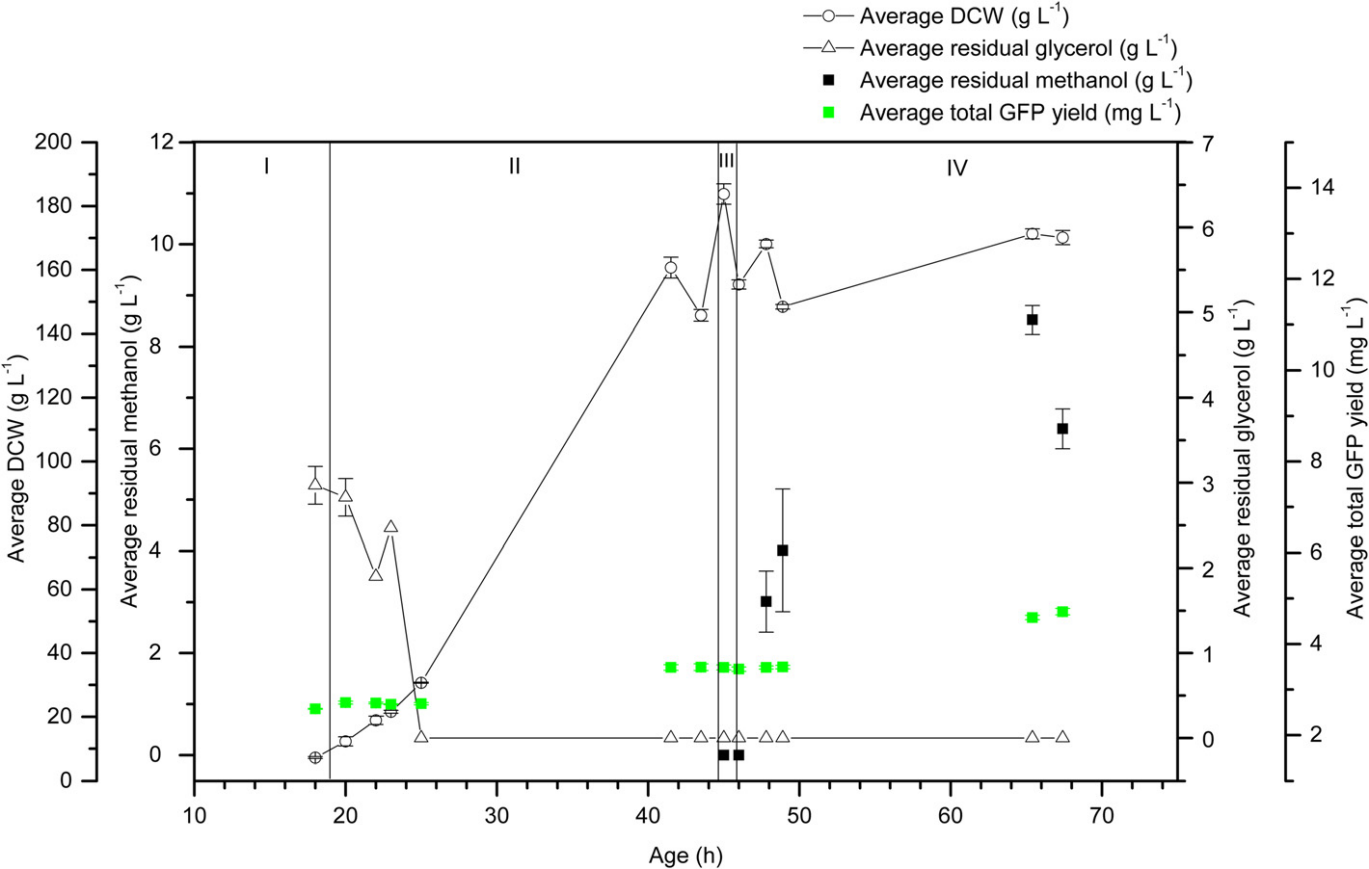
**Recombinant GFP is produced in the pre-induction phases of 1 L glycerol-grown** ***P. pastoris*** **bioreactor cultivations.** GFP yield (mg L^−1^; green squares) was measured in the culture supernatant in all phases of duplicate 1 L glycerol-grown *P. pastoris* cultures. The residual glycerol concentration (g L^−1^; white triangles) was at its highest during phase I, eventually dropping to zero in phase II. The DCW (white circles) for this cultivation reached a maximum of 175 g L^−1^. Residual methanol (g L^−1^; black squares) ranged from 3.01 to 8.52 g L^−1^. All measurements were made in triplicate and error bars represent the SEM.

In order to assess whether pre-induction expression was specific to hA_2a_R as the recombinant target protein, we examined the production of recombinant soluble GFP. Figure 3 shows the data for duplicate 1 L bioreactor cultivations with glycerol as the pre-induction carbon source. The residual glycerol concentration was at its highest during phase I, eventually dropping to zero in phase II: during the batch phase (I), glycerol was present at 3.0 g L^−1^ and the recombinant GFP yield was 2.6 mg L^−1^ prior to methanol addition. The GFP yield increased from 2.7 to 3.5 mg L ^−1^ in the fed-batch phase (II; the glycerol was consumed 3 h into this fed-batch phase), reaching a final yield of 4.7 mg L^−1^ in the induction phase (IV). Residual methanol ranged from 3.01 to 8.52 g L^−1^ and DCW reached a maximum of 175 g L^−1^.

### hA_2a_R and GFP are produced in the pre-induction phases of glucose-grown *P. pastoris* bioreactor cultivations

**Figure 4:**
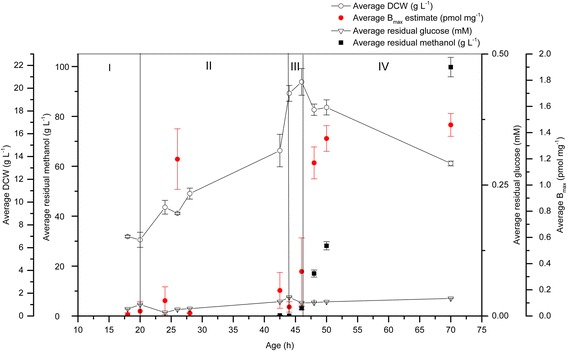
**Recombinant hA**_**2a**_ **R is produced in the pre-induction phases of 1 L glucose-grown** ***P. pastoris*** **bioreactor cultivations.** hA_2a_R binding activity (pmol mg^−1^; red circles) was measured in the culture supernatant in all the phases of a 1L glucose-grown *P. pastoris* cultivation. The residual glucose concentration (mM; white inverted triangles) ranged from 0.01-0.04 mM during the cultivation was not consumed completely by the cells. The residual methanol (g L^−1^; black squares) ranged from 0.20 to 99.76 g L^−1^. The DCW (g L^−1^; white circles) for this cultivation reached a maximum of 21 g L^−1^. All measurements were made in triplicate and error bars represent the SEM.

**Figure 5:**
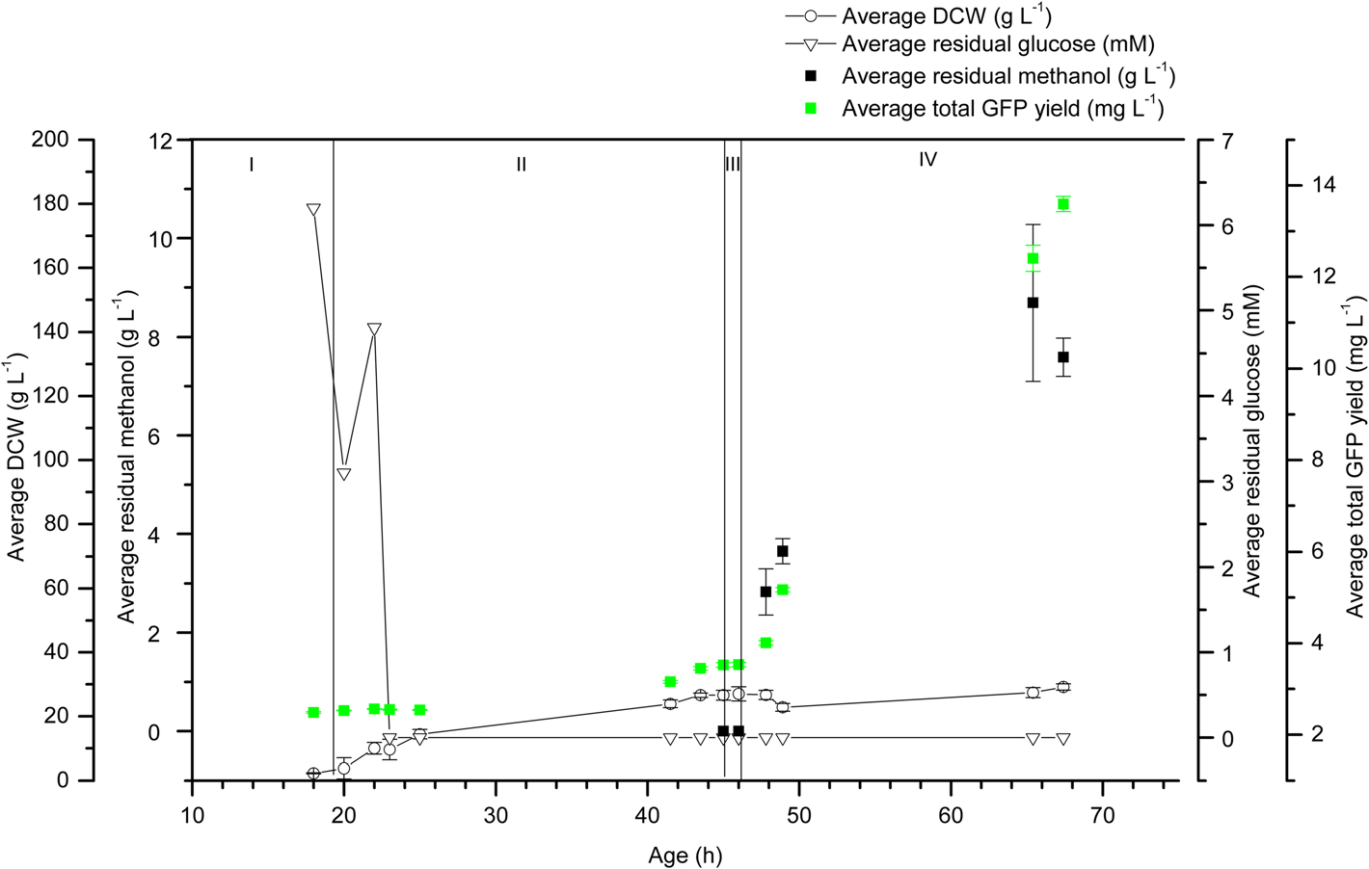
**Recombinant GFP is produced in the pre-induction phases of 1 L glucose-grown** ***P. pastoris*** **bioreactor cultivations.** GFP yield (mg L^−1^; green squares) was measured in the culture supernatant in all phases of duplicate 1 L glucose-grown *P. pastoris* cultivations. The residual glucose concentration (mM; white inverted triangles) was at its highest during phase I, eventually dropping to zero in phase II. The residual methanol (g L^−1^; black squares) ranged from 2.83 to 8.69 g L^−1^. The DCW (g L^−1^; triangles) for this cultivation reached a maximum of 30 g L^−1^. All measurements were made in triplicate. The error bars represent the SEM.

While glycerol de-represses *AOX* expression, glucose has been shown to repress *AOX* expression [19] even in the presence of methanol [20]. Duplicate cultivations were therefore analysed in which glucose replaced glycerol as the pre-induction carbon source in the recombinant production of both hA_2a_R (Figure 4) and GFP (Figure 5). For the production of hA_2a_R (Figure 4), the residual glucose concentration ranged from 0.01 to 0.04 mM; glucose was not fully consumed by the cells during the cultivation. However, when the residual glucose was very low during the fed-batch phase (phase II; 0.012 mM), the B_max_ was 1.2 pmol mg^−1^ which was similar to B_max_ values during methanol induction (phase IV; 1.17 to 1.46 pmol mg^−1^). The residual glucose also remained above zero during the transition phase (III, no glucose added). The hA_2a_R yield decreased between 26 and 46 hours (phase II and III) and then increased to 1.17 – 1.46 pmol mg^−1^ when methanol was added for the induction phase (IV). Methanol levels reached cytotoxic levels rapidly (99.76 g L^−1^) by the end of the cultivation which enabled us to confirm our earlier findings at pilot scale for glycerol-grown cultures (Table 1): we observed that for the glucose-grown cultures in Figure 4, pre-induction yields could be at least maintained after methanol induction, even in the presence of cytotoxic concentrations of methanol. The DCW reached a maximum of 21 g L^−1^ by the end of the cultivation. These results differed from the glycerol grown cells (Figure 1): the DCW weight produced by the glucose-grown cultivation was only 21 g L^−1^ when compared to greater than 100 g L^−1^ for the glycerol-grown cultivation. The hA_2a_R levels also differed: better yields were produced from the glycerol-grown cultivation (a maximum of 4.4 pmol mg^−1^ compared to a maximum of 1.46 pmol mg^−1^ at cytotoxic methanol levels, respectively).

For the production of GFP (Figure 5), the residual glucose concentration was at its highest during phase I, eventually dropping to zero in phase II: during the batch phase (I), when the glucose concentration was 6 mM, the yield of recombinant GFP was 2.5 mg L^−1^ prior to methanol addition. This increased from 2.5 to 3.5 mg L^−1^ in the fed-batch phase (II; the glucose was consumed 3 h into this fed-batch phase), reached a plateau in the transition phase (III) and then increased during the induction phase (IV) to a maximum value of 13.6 mg L^−1^. The residual methanol concentration ranged from 2.83 to 8.69 g L^−1^ and the DCW reached a maximum of 30 g L^−1^. Once the pre-induction carbon source was depleted, DCW values for glycerol-grown cells (Figure 3) were approximately 5 times higher than for glucose-grown cells in the equivalent phase. However, glucose-grown cells exhibited a higher specific productivity than glycerol-grown cells, producing 3 times the yield of GFP at the expense of their biomass yields.

### GFP is produced in both glycerol- and glucose- grown *P. pastoris* cultivations in the absence of a methanol induction step

**Figure 6:**
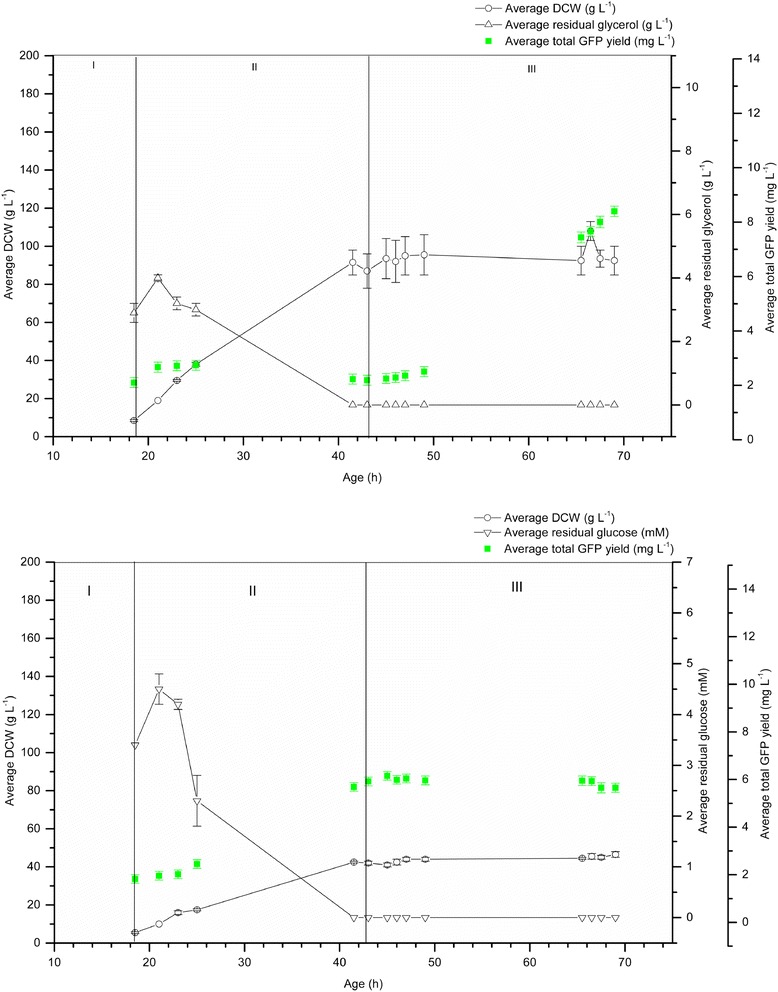
**Recombinant GFP is produced in methanol-free cultivations of** ***P. pastoris*** **grown on either glycerol or glucose as the sole carbon source.** GFP yield (mg L^−1^; green squares) was measured in the culture supernatant in all phases of two 1 L *P. pastoris* cultivations, one grown on glycerol (upper graph) and one grown on glucose (lower graph). The residual glycerol concentration (upper graph; g L^−1^; white triangles) was at its highest during phase I, eventually dropping to zero in phase II. The DCW (upper graph; g L^−1^; white circles) for this cultivation reached a maximum of 92.5 g L^−1^. The residual glucose concentration (lower graph; mM; white inverted triangles) eventually dropped to zero in phase II. The DCW (lower graph; g L^−1^; white circles) for this cultivation reached a maximum of 46.5 g L^−1^. All measurements were made in triplicate the error bars represent the SEM.

Since GFP was present in the pre-induction phases of both glycerol- and glucose-grown *P. pastoris* bioreactor cultivations (Figures 3 and 5), its production was analyzed in the absence of a methanol induction step (Figure 6). GFP yield was measured in the culture supernatant in all phases of two 1 L *P. pastoris* cultivations, one grown on glycerol and one grown on glucose (Figure 6). In these cultivations, the transition phase (III) was extended from 2 h to 30 h. During the batch phase (I) of the glycerol-grown culture (Figure 6; upper graph), glycerol was present at 2.9 g L^−1^ and the recombinant GFP yield was 2.1 mg L^−1^ in line with the earlier glycerol-grown cultivations (2.6 mg L^−1^ prior to methanol addition, Figure 3). The total GFP yield remained stable in the fed-batch phase (II) and reached a final yield of 8.4 mg L^−1^ at the end of the cultivation. This was higher than the yield achieved in the corresponding induced cultures (4.7 mg L^−1^ at the end of phase IV; Figure 3) indicating the negative impact of the methanol feed on total yield. The DCW for the methanol free cultivation reached a maximum of 92.5 g L^−1^ (Figure 6; upper graph) in contrast to that of 175 g L^−1^ for the corresponding induced culture (Figure 3).

During the batch phase (I) of the glucose-grown culture (Figure 6; lower graph), glucose was present at 3.4 mM and the recombinant GFP yield was 1.8 mg L^−1^, which was lower than that for the earlier glucose-grown cultivation (2.5 mg L^−1^; Figure 3). The total GFP yield increased in the fed-batch phase (II), remained stable in the transition phase and reached a final yield of 5.6 mg L^−1^ at the end of the cultivation. This was lower than the yield achieved in the corresponding induced culture (13.6 mg L^−1^ at the end of phase IV; Figure 3) indicating the positive impact of the methanol feed on total yield. The DCW reached a maximum of 46.5 g L^−1^ in contrast to that of 30 g L^−1^ for the corresponding induced culture (Figure 5).

### hCGRP-RCP-GFP is also produced in pre-induction phases of glycerol-grown *P. pastoris* bioreactor cultivations, while pre-induction yields of five proteins are not detected in shake flask cultivations

To examine whether these findings were applicable to other target proteins, we examined the pre-induction phases of glycerol-grown bioreactor cultivations producing hCGRP-RCP-GFP [21]: hCGRP-RCP-GFP was detected in the pre-induction phases (Table 2, Additional file 1). In contrast, shake-flask cultivations of GFP [22], HRP, hCD81 [23], hCD82 [23] and human claudin-1 [23] showed no recombinant protein production in the pre-induction phases (Table 2, Additional file 1).

**Table 2:** **Functional recombinant protein is present in the pre-induction phases of** ***P. pastoris*** **cultures when cultivated in bioreactors, but not in shake-flasks**

**Protein**	**Bioreactor**		**Shake-flask**	
	***Pre-induction expression in glycerol-grown cells?***	***Pre-induction expression in glucose-grown cells?***	***Pre-induction expression in glycerol-grown cells?***	***Pre-induction expression in glucose-grown cells?***
***Integral membrane proteins***				
hA_2a_R	Y (Figures 1 and 2)	Y (Figure 4)	N [18]	–
CD81	–	–	N (Additional file 1: Figure S1)	–
CD82	–	–	N (Additional file 1: Figure S1)	N (Additional file 1: Figure S1)
Claudin-1	–	–	N (Additional file 1: Figure S1)	N (Additional file 1: Figure S1)
***Peripheral membrane protein***				
hCGRP-RCP-GFP	Y (Table S1)	–	–	–
***Soluble proteins***				
GFP	Y (Figures 3 and 6A)	Y (Figures 5 and 6B)	N (Additional file 1: Table S2)	N (Additional file 1: Table S2)
HRP	–	–	N (Additional file 1: Table S3)	–

The presence (Y) or absence (N) of recombinant protein was determined in bioreactor and shake-flask cultivations for glycerol- and glucose-grown cells as indicated; the evidence supporting these designations is given in parentheses and refers to figures within the text or in supplementary files; “–” indicates that the experiment was not done.

## Discussion

It is established that both the concentration and rate of addition of methanol to *P. pastoris* cultures can substantially affect recombinant protein yields [20,24–28]. In contrast, the impact of the pre-induction phases of *P. pastoris* cultures on yield has not been examined systematically in either bioreactor or shake-flask formats.

A significant finding emerging from this work is that an integral membrane protein (hA_2a_R), a soluble protein (GFP) and a peripheral membrane protein (hCGRP-RCP-GFP) are produced prior to induction with methanol in bioreactor cultivations of *P. pastoris*. This is consistent with a previous report by Singh and colleagues who noted pre-induction hA_2a_R binding activity in glycerol-grown bioreactor cultivations of *P. pastoris*, an observation that was not further elaborated upon [14]. In a different bioreactor study by Çelik and colleagues, recombinant hEPO appeared to be produced prior to the onset of the methanol feed, but this was not discussed by the authors [24]. In contrast, pre-induction hA_2a_R binding activity was not apparent in shake-flasks using complex medium in studies by Singh and colleagues [15] or by Fraser [18]. In agreement with this, we show that GFP, HRP, hCD81, hCD82 and human claudin-1 are not detected in the pre-induction phases of shake-flask cultivations. The reason for this is currently unknown; we suspect it may be a function of the different oxygenation of cultures in the two formats and/or the different media used (complex in shake-flasks and minimal in bioreactors) [29].

The demonstration of recombinant protein synthesis prior to induction is especially noteworthy because glycerol, glucose, ethanol and acetate have all been shown to support growth of *P. pastoris* cells without inducing the *AOX* promoter [19]; our systematic study demonstrates that recombinant hA_2a_R and GFP can be detected in both glycerol- and glucose-grown bioreactor cultivations prior to methanol induction, confirming earlier suggestions of leaky expression from *AOX* promoters. Our data therefore suggest that the productivity of *AOX*-dependent bioprocesses is not solely reliant on induction by methanol and that in order to maximize total yields, the impact on specific productivity of pre-induction phase cultivation should be optimized.

Our data demonstrate that methanol induction does not necessarily increase the pre-induction specific yield, measured in the transition phase, for cells initially cultured on glycerol. The total yield of hA_2a_R was at least maintained once the methanol feed commenced during the induction phase (Figure 1, Table 1), but the increase in total yield was not substantial, being only approximately double that of the pre-induction yield (Figure 1, Table 1). A subsequent glycerol-grown *P. pastoris* cultivation also produced recombinant GFP in the pre-induction phases, with similar results following induction with methanol (Figure 3). These increases in yield are similar to those seen by Çelik and colleagues in their hEPO cultivations [24].

Pre-induction hA_2a_R yields were maintained even under cytotoxic methanol concentrations (Table 1 and Figure 4). Cunha and colleagues [30] and Schenk and colleagues [31] demonstrated that residual methanol concentrations between 2 g L^−1^ and 3.5 g L^−1^ were optimal for soluble protein production, while methanol concentrations between 3.7 g L^−1^ and 20 g L^−1^ were cytotoxic and inhibited growth [30,31], consistent with our findings. We previously reported that residual methanol levels below 2.98 g L^−1^ gave optimal yields of recombinant GFP (3.74 g) when following a mixed 60% methanol and 40% sorbitol induction regime. In contrast, a 100% methanol induction regime, where the residual methanol reached a maximum level of 182.25 g L^−1^, gave a poor recombinant GFP yield (0.09 g) [27].

Previously, we have demonstrated that high yields of recombinant GFP (3.74 g) but low biomass yields (OD_595_ = 64) were produced when *P. pastoris* was grown at a low induction phase growth rate (μ = 0.006 h ^−1^). In contrast, a higher induction phase growth rate (μ = 0.015 h ^−1^) resulted in a higher biomass yield (OD_595_ = 74), but a lower total GFP yield (0.98 g) [27]. Çalık and colleagues [32] applied three different growth rates in the methanol induction phase of *P. pastoris* cultures expressing soluble human growth hormone: 0.02, 0.03 and 0.04 h ^−1^ using a mixed sorbitol and methanol feed. The highest biomass yield (48 g L^−1^) was obtained at a growth rate of 0.04 h ^−1^, but the highest yield of recombinant human growth hormone (270 mg L^−1^) was produced at 0.03 h ^−1^. The authors suggested that this might be due to the lower growth rate producing lower biomass and therefore fewer extracellular proteases being present in the cultivation. Similar trends were also observed for GFP, hEPO, human serum albumin, heavy-chain fragment C of *Botulinum* neurotoxin serotype A and avidin [24,26,27,33].

Glucose has previously been shown to support growth of *P. pastoris*, but at a lower specific growth rate and yielding lower amounts of biomass than that achieved by cells grown on glycerol [34]. Following induction with methanol, DCW values for glycerol-grown cells producing GFP were approximately 5 times higher than for glucose-grown cells in the equivalent pre-induction phase (Figures 3 and 5), although the glucose-grown cells produced 3 times the GFP yield of the glycerol-grown cells (Figures 3 and 5). Notably, the methanol feed had a positive impact on yield for glucose-grown cells, but a negative impact on yield for glycerol-grown cells (Figures 3, 5 and 6). For hA_2a_R produced in glucose-grown cultures (Figure 4), methanol also had a positive impact on yield. However, the yields compared to the glycerol-grown cells were lower, which was in contrast to GFP yields produced in glucose-grown cells.

At pilot scale, the methanol feed influenced the biomass yield and particularly increased the total hA_2a_R yield in the low methanol cultivation compared to the high methanol cultivation (Table 1). Notably, a similar increase in total membrane protein yield has previously been reported to increase total volumetric yields of GPCRs in a respiratory yeast strain of *Saccharomyces cerevisiae* [35]. As a consequence of increased total membrane protein yield, the yield per unit volume of the low methanol cultivation was approximately 40 times higher than shake flask cultivations of the same hA_2a_R construct [18]; total membrane protein yields from shake flasks are 200 mg [18] compared with almost 8,000 mg in bioreactors (Table 1). This yield improvement compares very favourably with that achieved by Singh and colleagues using an optimized hA_2a_R construct under broadly similar bioprocess conditions to our study [15]: on transferring from shake flasks to bioreactors a yield improvement of approximately 25 times was achieved on account of both an increase in specific productivity (25 pmol mg^−1^ in a shake flask; 100 pmol mg^−1^ in a bioreactor) and biomass yield (OD_600_ = 13 in a shake flask; OD_600_ = 80 in a bioreactor). Overall, our data suggest that specific productivity may not necessarily be solely dependent on methanol induction and can be increased at the expense of improvements in biomass yields in the methanol induction phase.

## Conclusions

Production of hA_2a_R, GFP and hCGRP-RCP-GFP was detected in bioreactors prior to methanol induction in glycerol- and glucose-grown cells; in contrast, GFP, HRP, hCD81, hCD82 and human claudin-1 were not detected in the pre-induction phases of shake-flask cultivations. In bioreactors, the transition phase yield was not necessarily increased following methanol induction and was maintained even at cytotoxic methanol concentrations. Using glucose as a pre-induction carbon source, specific productivity could be increased at the expense of improvements in biomass yield, with methanol having a positive impact on these cells. These data provide a platform to further optimize the production of recombinant proteins, and especially GPCRs [7] as part of modern drug discovery programmes.

## Methods

### Yeast strains and culturing conditions

*P. pastoris* strains X33 hA_2a_R [18], X33 GFP [22,29], SMD1163 hCGRP-RCP-GFP [21], CBS 7435 Mut^s^ HRP, X33 hCD81 [23], X33 hCD82 [23] and X33 human claudin-1 [23] were used in all experimental procedures using previously-described culture conditions. To generate a seed culture for the bioreactor studies or to establish a shake-flask culture, a single, freshly-streaked colony was used to inoculate 5 mL YPD medium (2% peptone, 1% yeast extract, 2% glucose), and this was incubated at 30°C in a shaking incubator for 2 days. The 5 mL YPD pre-culture was then used to inoculate 50 mL YPD in a 250 mL baffled shake-flask, and this was incubated at 30°C in a shaking incubator overnight to an OD_600_ of 15. For bioreactor cultivations, these cells were then used to inoculate 200 mL FM22 minimal medium, which was composed of (g L^−1^) KH_2_PO_4_, 42.9; (NH_4_)_2_SO_4_, 5; CaSO_4_·2H_2_O, 1.0; K_2_SO_4_, 14.3; MgSO_4_·7H_2_O, 11.7; and glycerol, 10 with *Pichia* trace minerals 4 (PTM_4_) salt solution composed of (g L^−1^) CuSO_4_·5H_2_O, 2.0; NaI,0.08; MnSO_4_·H_2_O, 3.0; Na_2_MoO_4_·2H_2_O, 0.2; H_3_BO_3_, 0.02; CaSO_4_·2H_2_O, 0.5; CoCl_2_, 0.5; ZnCl_2_, 7; FeSO_4_·7H_2_O, 22; biotin, 0.2; 1 ml of concentrated H_2_SO_4 ._ These bioreactor seeder flasks were incubated at 30°C and 220 rpm over-night to an OD_600_ of 30.

### 1 L bioreactor cultures

For the glycerol cultivations, 1 L FM22 medium supplemented with 0.5 mL J673A (Struktol), 4 mL PTM_4_ salts, 0.8 mL 10 g L^−1^ biotin and 10 g 100% glycerol was transferred to a 2 L (total volume) jacketed glass bioreactor (Applikon Biotechnology Ltd). Culture temperature was maintained at 30°C and pH at 5. The maximum agitation rate was 1,250 rpm. The air flow rate was 2 L min^−1^ and the DO set point was 30%. The end of the glycerol batch phase was indicated by a spike in DO to 100%. A glycerol (50% v/v aqueous glycerol) fed-batch phase was maintained for 40 h at a flow rate of 14 mL min^−1^. For the methanol induced cultivations, the transition phase was maintained for 2 h and the temperature was lowered to 22°C for cultivations producing hA_2a_R but remained at 30°C for the GFP cultivations. The induction phase comprised feeding 50% (v/v) aqueous methanol at 2.04 mL min^−1^ for 40 h. For the glucose cultivations, 1 L FM22 medium supplemented with 0.5 mL J673A (Struktol), 4 mL PTM_4_ salts, 0.8 mL 10 g L^−1^ biotin and 10% glucose was transferred to a 2 L (total volume) jacketed glass bioreactor (Applikon Biotechnology Ltd). Culture temperature was maintained at 30°C and pH at 5. The maximum agitation rate was 1,250 rpm. The end of the glucose batch phase was indicated by a spike in DO to 100%. A glucose (10%) fed-batch phase was maintained for 40 h at a flow rate of 14 mL min^−1^. For the methanol-free cultivations, the conditions remained the same as in the methanol induced cultivations for the batch and fed-batch phases. At the transition phase, the glycerol or glucose feed was stopped, the temperatures changed (22°C for the hA_2a_R cultivation and 30°C for the GFP cultivation) and the cultivations were allowed to run for a further 26 h without any methanol feed or any other carbon source. Bioreactor control was via an Applikon ADI1010 control unit.

### 10 L bioreactor cultures

10 L FM22 medium supplemented with 1 mL P2000 (Sigma-Aldrich), 40 mL PTM_4_ salts and 8 mL 10 g L^−1^ biotin were transferred into each of two 30 L (total volume) jacketed steel bioreactors (Biostat C, Sartorius Ltd). Culture temperature was maintained at 30°C and pH at 5. The maximum agitation rate was 1,500 rpm. For both cultivations, the end of the glycerol batch phase (phase I) was indicated by a spike in DO to 100%. A glycerol fed-batch phase (phase II) was then maintained for 40 h by employing an exponential feed rate of 50% aqueous glycerol (v/v) at 4 g L^−1^ h^−1^ increasing exponentially first at a rate of 0.15 h^−1^ for 10 h and subsequently at a rate of 0.03 h^−1^. The first section of the transition phase (phase IIIA; starvation), during which no further carbon source was fed into the bioreactor, was maintained for 1 h. The second section of the transition phase (phase IIIB) comprised a further 1 h, where a constant methanol feedstock (50% (v/v) aqueous methanol) was applied at 8 g L^−1^ h ^−1^. The culture temperature was lowered to 22°C and an exponential methanol (50% (v/v) aqueous methanol) feed profile was applied by exponentially increasing the feed rate at 0.01 h^−1^ (low methanol cultivation) or 0.03 h^−1^ (high methanol cultivation). The induction phase continued for 40 h.

### Sampling

Samples (1 mL) were taken for optical density measurements at 600 nm (OD_600_). For dry cell weight measurements, 1.5 mL culture was sampled in triplicate, placed in pre-weighed tubes and centrifuged at 5,000 × g for 5 min. The supernatant was removed and stored at −20°C for residual glycerol and/or residual methanol analysis and the tubes were placed in a 100°C oven with the lids open and dried overnight. The dried tubes were moved to a desiccator for 2–3 days and then weighed on a microbalance. The dry cell weights were reported as g L ^−1^ (g DCW per L of culture).

### Residual glycerol analysis

Residual glycerol in the culture supernatants was analysed spectroscopically at 340 nm using a glycerol quantitation kit (r-biopharm, Roche) according to the manufacturer’s protocol. Triplicate determinations were performed for each sample.

### Residual glucose analysis

Residual glucose in the culture supernatants was analysed using an Accu-Chek Active Glucose analyser (Roche Diagnostics) or by Amplex® Red Glucose/Glucose Oxidase Assay Kit (Life Technologies) according to the manufacturer’s protocol. Triplicate determinations were performed for each sample.

### Residual methanol analysis

Residual methanol in the culture supernatants was analysed using a Thermo Scientific FOCUS Gas Chromatograph. Appropriately-diluted culture supernatants (1 μL) were injected in duplicate and the methanol peaks integrated using QuanLab® software. The mean value of the integrated peak area was used to estimate the residual methanol within the sample by comparison with methanol standards. Triplicate determinations were performed for each sample.

### Preparations of membrane fractions

Harvested cells producing recombinant hA_2a_R, hCD81, hCD82 or human claudin-1 were re-suspended in 2 mL breaking buffer (50 mM Na_2_HPO_4_, 2 mM EDTA, 100 mM NaCl, 5% glycerol w/v, pH 7.4) per gram of wet cell weight and disrupted at 30,000 psi for 20 min using an Avestin® C3 pressure homogeniser. Breaking efficiency was typically > 90% as determined by light microscopy. The samples were clarified by centrifugation at 10,000 × g, 4°C for 30 min and total membranes recovered from the supernatant at 100,000 × g, 4°C for 1 h. Total membranes were re-suspended in 10 mL Buffer A (20 mM HEPES, 50 mM NaCl, 10% glycerol, pH 7.0) per gram of total membrane. Protein concentration was determined using a BCA assay (Sigma-Aldrich) using BSA as a protein standard. 0.5 mL aliquots of membrane fraction were stored at −80°C prior to further analysis. Analysis by immunoblot was done as described previously [23].

### Radio-ligand binding assays

Membrane bound hA_2a_R was analysed using a single-point saturation radio-ligand binding assay. Membranes (0.5 mg mL^−1^) supplemented with 0.1 U of adenosine deaminase (Sigma-Aldrich) were incubated with 10 nM [^3^H]-ZM241385 (American Radiolabelled Chemicals UK Ltd) for 1.5 h at 30°C. 10 nM of [^3^H]-ZM241385 is expected to saturate the receptor [18]. To assay non-specific binding, 1 μM ZM241385 (Tocris) was added to the incubations. Assays were terminated by centrifugation at 14,000 rpm in a bench-top centrifuge for 5 min at 4°C. The supernatant was discarded and the pellets were washed with water and then solublilised with Soluene® (PerkinElmer) overnight. Solubilised pellets were added to scintillation fluid and counted in a scintillation counter (Packard 1600TR Liquid Scintillation Analyser) to determine the amount of bound radio-ligand. Triplicate determinations were performed for each sample. B_max_ values were calculated from the Langmuir occupancy equation and reported as pmol hA_2a_R per mg of total membrane protein [18], as determined by BCA assay.

### Recombinant GFP analysis

Supernatants (100 μL) from cultures secreting GFP were assayed for GFP fluorescence using a Spectramax Gemini XS® plate reader with an excitation wavelength (λ_exe_) of 397 nm and emission wavelength (λ_em_) of 506 nm at 25°C. Triplicate determinations were performed for each sample. All samples and blanks were buffered to pH >7.0 using 50 μL 1 M potassium phosphate pH 8.0. To determine the concentration of GFP in each of the samples, a recombinant GFP standard (Vector Laboratories Ltd) was used to construct a standard curve relating GFP fluorescence to protein concentration, as previously described [22].

### Recombinant CGRP-RCP-GFP analysis

Recombinant CGRP-RCP-GFP was analysed from culture supernatants by immunoblot as described previously [23].

### Recombinant HRP analysis

Supernatants (25 μL) from cultures secreting HRP were assayed for HRP using a 2 Component ABTS Peroxidase Substrate system (Kirkegaard Perry Laboratories) containing ABTS [2,2′-azino-di-(3-ethylbenzthiazoline-6-sulfonate)] substrate and hydrogen peroxide. This liquid substrate system produces a blue-green coloured product when reacted with peroxidase present in the test samples. 25 μl of culture supernatant was mixed with 150 μl of ABTS-H_2_O_2_ in a 96 well plate and the increase in absorbance of the end product (after 10 minutes of incubation at room temperature) was read at 405 nm using a spectrophotometer (Multiskan GO, Thermo Scientific). To determine the concentration of HRP in each of the samples, a purified HRP standard (Sigma-Aldrich) was used to generate a standard curve relating peroxidase activity to protein concentration.

### Graphical presentation

Graphs were constructed using OriginPro 9.1® software.

## Additional file

Additional file 1:
**Supplementary data for bioreactor and shake-flask cultivations. Table S1.** hCGRP-RCP-GFP is present in all phases of 1 L glycerol-grown *P. pastoris* bioreactor cultivations. **Table S2.** GFP is not produced in the pre-induction phases of glycerol- or glucose-grown *P. pastoris* shake-flask cultivations. **Table S3.** HRP is not produced in the pre-induction phases of glycerol-grown *P. pastoris* shake-flask cultivations. **Figure S1.** hCD81, hCD82 and human claudin-1 are not produced in the pre-induction phases of *P. pastoris* shake-flask cultivations as determined by immunoblot.
