# Supplementary material for: Functional recombinant protein is present in the pre-induction phases of Pichia pastoris cultures when grown in bioreactors, but not shake-flasks
Source: Microb Cell Fact. 2014 Sep 4;13:127. doi: 10.1186/s12934-014-0127-y (PMC4159547; doi:10.1186/s12934-014-0127-y)
Supplement: Additional file 1: — Supplementary data for bioreactor and shake-flask cultivations. Table S1. hCGRP-RCP-GFP is present in all phases of 1 L glycerol-grown P. pastoris bioreactor cultivations. Table S2. GFP is not produced in the pre-induction phases of glycerol- or glucose-grown P. pastoris shake-flask cultivations. Table S3. HRP is not produced in the pre-induction phases of glycerol-grown P. pastoris shake-flask cultivations. Figure S1. hCD81, hCD82 and human claudin-1 are not produced in the pre-induction phases of P. pastoris shake-flask cultivations as determined by immunoblot. [file 12934_2014_127_MOESM1_ESM.pdf]

Supplementary data for bioreactor cultivations

Table S1 – hCGRP-RCP-GFP is present in all phases of 1 L glycerol-grown *P. pastoris* bioreactor cultivations. Duplicate 1 L bioprocesses were analysed for the production of hCGRP-RCP-GFP. Fluorescent measurements were made in triplicate for each culture. The standard error of the mean (SEM) is reported for each time point.

Phase	Age (h)	hCGRP-RCP-GFP yield (mg/L)	SEM
I	0	0	0
II	27	87	1.1
III	46	136	2.9
IV	52.5	154	0.8
IV	75	143	0.9

Supplementary data for shake-flask cultivations

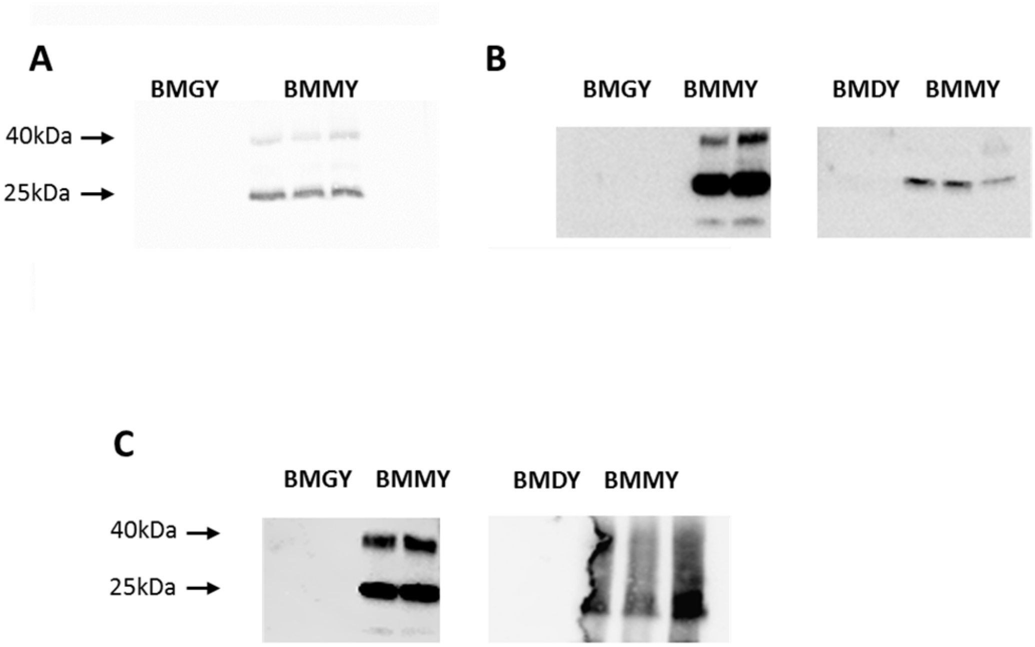

Figure S1 – hCD81, hCD82 and human claudin-1 are not produced in the pre-induction phases of *P. pastoris* shake-flask cultivations as determined by immunoblot. 50 mL shake-flask cultivations were analysed for the production of (A) hCD81, (B) hCD82 and (C) human claudin-1. Measurements were made in BMGY (glycerol-containing medium), BMDY (glucose-containing medium) and BMMY (methanol-containing medium) as indicated. Proteins were detected using a rabbit anti-His$_6$ monoclonal antibody (Serotec).

Table S2 –GFP is not produced in the pre-induction phases of glycerol- or glucose-grown *P. pastoris* shake-flask cultivations. Triplicate shake-flask cultures were analysed for the production of GFP. Measurements were made in BMGY (glycerol-containing medium), BMDY (glucose-containing medium) and BMMY (methanol-containing medium). Proteins were detected by fluorimetry.

Phase	Age (h)	GFP yield (μg/mL)	SEM
BMGY	17.5	0	0
BMGY	24	0	0.6
BMMY	41.5	0.7	0.8
BMMY	48	2.2	0.8
BMMY	68.5	18.2	3.2
BMDY	17.5	0	0
BMDY	24	0	0
BMMY	41.5	1.5	0.3
BMMY	48	1.4	0.2
BMMY	68.5	4.2	0.5

Table S3 –HRP is not produced in the pre-induction phases of glycerol-grown *P. pastoris* shake-flask cultivations. Duplicate shake-flask cultures were analysed for the production of HRP. Measurements were made in BMGY (glycerol-containing medium) and BMMY (methanol-containing medium). Proteins were detected by colorimetric assay at 405nm.

Phase	Age (h)	HRP yield (μg/mL)
BMGY	18	0
BMMY	0	0
BMMY	24	0.4
BMMY	48	1.3
BMMY	64	2.4
